# *Nocardia vulneris*: a rare pathogen in actinomycetoma – a case report and review of the literature

**DOI:** 10.1093/skinhd/vzaf041

**Published:** 2025-06-25

**Authors:** Teerapong Rattananukrom, Roberto Arenas, Yosbeli Ramírez, Ana Luz Ely Guevara-Cerritos, Rigoberto Hernandez-Castro

**Affiliations:** Division of Dermatology, Department of Medicine, Faculty of Medicine Ramathibodi Hospital, Mahidol University, Bangkok, Thailand; Mycology Section, ‘Dr Manuel Gea Gonzalez’ General Hospital, Mexico City, Mexico; Residente del Primer año de Dermatología, Universidad Evangélica de El Salvador, San Salvador, El Salvador; Dermatóloga Adscrita at Hospital Nacional ‘Dr Juan José Fernández Zacamil’, Centro Dermatológico, San Salvador, Mejicanos, El Salvador; Departamento de Ecología de Agentes Patógenos, ‘Dr Manuel Gea Gonzalez’ General Hospital, Mexico City, Mexico

## Abstract

Actinomycetoma is a chronic, progressive bacterial infection characterized by granuloma formation, with *Nocardia vulneris* being a rare causative agent. A 32-year-old healthy man from El Salvador presented with painless nodules, scars and discharging sinus tracts on his back. Examination of the exudate revealed small white grains, and *Nocardia* species were isolated through culture. Molecular identification of *N. vulneris* was confirmed via 16S rDNA gene amplification and sequencing. The patient was treated with trimethoprim–sulfamethoxazole and dapsone, resulting in significant clinical improvement after 6 months. He continues on this treatment regimen. This case highlights the rarity of *N. vulneris* mycetoma at an unusual anatomical site and demonstrates the effectiveness of combined trimethoprim–sulfamethoxazole and dapsone therapy.

## Teaser

Actinomycetoma is a chronic granulomatous bacterial infection, with *N. vulneris* being a rare associated pathogen. Therapeutic strategies for *N. vulneris* are primarily based on case reports, with TMP-SMX and dapsone proving effective for localized infections. This case underscores the rarity of *N. vulneris* mycetoma, which is a first report in Central America and highlights the efficacy of combined TMP-SMX and dapsone therapy.

Mycetoma is a chronic, progressive granulomatous infection that can be caused by fungi (eumycetoma) or bacteria (actinomycetoma). Clinically, mycetoma presents with tumour-like swellings, multiple sinuses and grains. *Nocardia brasiliensis* and *Nocardia asteroides* are the most frequently reported species responsible for actinomycetoma globally. However, other less common species, such as *Nocardia otitidiscaviarum*, *Nocardia transvalensis*, *Nocardia mexicana*, *Nocardia takedensis*, *Nocardia harenae* and *Nocardia vulneris* have also been implicated. *Nocardia vulneris* is a species within the genus *Nocardia*, comprising aerobic, Gram-positive, filamentous bacteria widely distributed in soil, water and decaying organic matter. Identified as a distinct species based on genetic and phenotypic characteristics, *N. vulneris* is a rare pathogen within this genus. Like other *Nocardia* species, it is an opportunistic pathogen that predominantly affects immunocompromised individuals but can also infect immunocompetent hosts. *Nocardia vulneris* has been linked to actinomycetoma – a chronic, localized, granulomatous infection – and disseminated infections.^1^ This report aims to present the first documented case of mycetoma caused by *N. vulneris* in Central America, occurring on the back of an immunocompetent patient, and to highlight successful treatment with a combination therapy regimen.

## Report

A 32-year-old healthy man from El Salvador, presented with a firm painless plaque 20 cm diameter composed of a lesion with a nodular aspect, and purulent draining sinuses on the right side of his back, persisting for 18 months. Over time, the condition progressed, resulting in painless inflammatory lesions with draining sinuses. The patient, employed in an egg factory, had no significant medical history and denied any underlying disease or current use of medications or herbal supplements. The patient had a history of traumatic inoculation due to his work, but he did not recall a specific incident because he frequently experiences minor injuries including the fingers, toes, arms and back. In addition, the patient did not exhibit any systemic symptoms or specific signs indicating involvement of the lungs, neural tissue or bone. He had no fever, headaches or back pain. He had no special dietary preferences, did not smoke and occasionally consumed alcohol.

**Figure 1:**
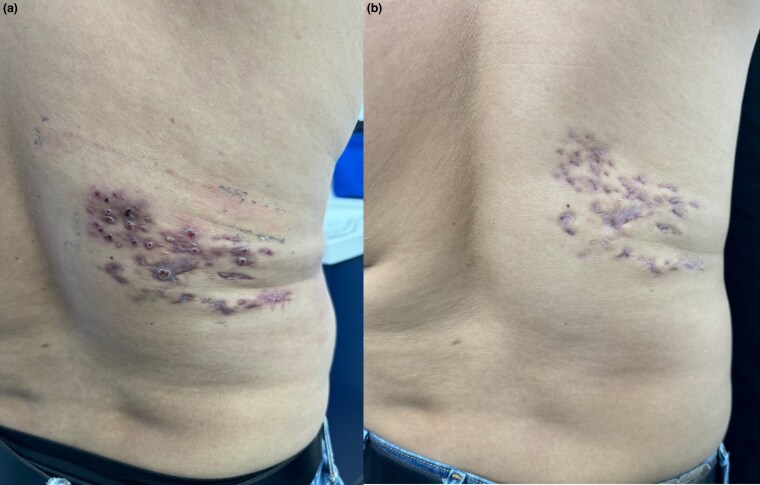
(a) A 32-year-old man with painless nodular lesions and discharging sinuses on the right side of the back. (b) Treatment with trimethoprim–sulfamethoxazole and dapsone led to clinical improvement after 6 months of follow-up.

**Figure 2:**
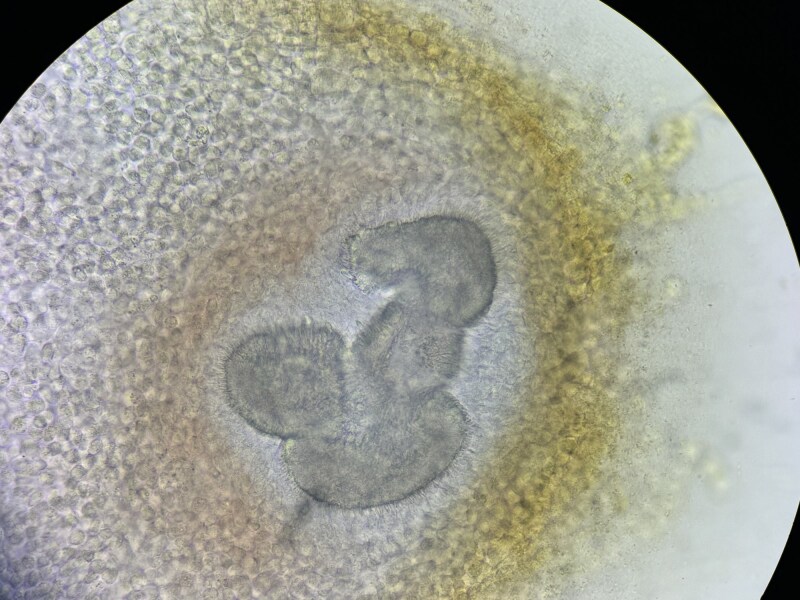
Direct examination of small white grains (100–200 µm) under KOH preparation (×40) showed compact, branching filaments characteristic of *Nocardia* species, consistent with white-grain mycetoma.

**Figure 3:**
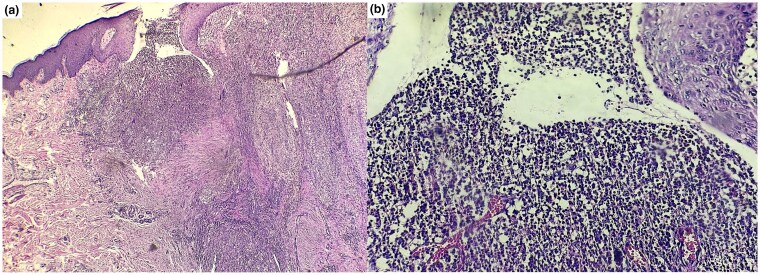
Histopathological examination of actinomycetoma revealed suppurative granulomatous inflammation. (a) Inflammatory infiltrates, including neutrophils, lymphocytes and plasma cells, were observed along with areas of fibrosis (H&E, ×10). (b) Higher magnification (H&E, ×40) confirmed the absence of grains in the tissue. H&E, haematoxylin and eosin.

On physical examination, the patient exhibited painless numerous erythematous lesions and discharging sinuses on the right side of his back (Figure 1a). Direct examination of the white grains using a potassium hydroxide preparation (×40) revealed microscopic small white grains measuring 100–200 µm (Figure 2). An incisional biopsy of the back lesion, stained with haematoxylin and eosin (H&E), showed suppurative granulomatous inflammation. Inflammatory infiltrates, including neutrophils, lymphocytes and plasma cells, were observed along with areas of fibrosis (H&E, ×10). However, higher magnification (H&E, ×40) confirmed the absence of grains in the tissue (Figure 3).

A Sabouraud dextrose agar culture, incubated at room temperature (25–27 °C), produced white light cream and chalky colonies after 10 days. These colonies measured 1–2 mm in diameter, appearing singly or in bundles, and became whitish and tortuous with prolonged incubation. Laboratory tests revealed no anaemia (haemoglobin, 12 g dL^–1^) and no leucocytosis (white blood cell count, 8 × 10³ cells μL^–1^). No elevations were noted in the aspartate aminotransferase level (30 U L^–1^; normal range 5–34 U L^–1^) or the alanine aminotransferase level (50 U L^–1^; normal range 0–55 U L^–1^). The creatinine level was 0.91 mg dL^–1^ (normal range 0.55–1.02 mg dL^–1^). The glucose-6-phosphate dehydrogenase enzyme activity test was within normal limits, as was the lipid profile. Additionally, the haemoculture was negative. Chest and lumbosacral spine X-ray was normal, with no osteolytic lesions detected. Magnetic resonance imaging was not performed due to the absence of back pain, neuropathy or motor weakness.

The molecular identification was performed by sequencing the 16S rDNA gene. Primers (5′-GGATCCTTTTGATCCTGGCTCAGGAC-3′ and 5′-ACTTGACGTCGTCCCCACCTTCCTC-3′) were designed based on the 16S rDNA sequence of *N. wallacei*, American Type Culture Collection 49872, formerly *N. asteroides* (GenBank accession number: AY191251). A polymerase chain reaction (PCR) product of 1400 bp was amplified, purified and sequenced in both directions. The sequence was edited using the Vector NTI program, and a homology search in the GenBank database (nucleotide BLAST) revealed a 100% identity with *N. ­vulneris* strains LPB4002 and W9851. The entire sequence of *N. vulneris* strain GEA-60 was deposited in the GenBank sequence database under accession number 0870.

The patient was diagnosed with actinomycetoma caused by *N. vulneris*. Treatment consisted of a daily regimen of trimethoprim–sulfamethoxazole (TMP-SMX 800/160 mg, two double-strength tablets every 12 h) and dapsone (100 mg daily). After 6 months of follow-up, the patient showed significant improvement while continuing the same treatment (Figure 1b). During treatment, the patient reported no side effects from TMP-SMX or dapsone, including severe haemolysis, hypersensitivity reactions or bone marrow suppression. Over the 6-month follow-up period, no neurological symptoms were observed.

## Discussion

A total of 19 494 mycetoma cases were identified from 1876 to 2019 across 102 countries. Most cases have been reported in Mexico, India and Sudan, where mycetoma is extensively studied. New geographical areas of emergence include the USA, Venezuela, Italy, China and Australia, highlighting that mycetoma occurs even beyond tropical regions.^2^ Mycetoma, a World Health Organization (WHO)-recognized neglected tropical disease, is caused by filamentous fungi or bacteria. Despite being preventable and treatable in its early stages, it leads to high morbidity and socioeconomic burden. It typically presents as woody swelling with discharging sinuses and grains, but may also occur without sinuses. If untreated, it can invade bone and muscle, causing permanent disability.^3^

Eumycetoma typically presents as a slowly progressive, tumour-like swelling with black discharging grains. In contrast, actinomycetoma progresses more rapidly and produces yellow or white grains. Eumycetoma grains are black or brown, firm (0.2–5 mm) and composed of branching, septate fungal hyphae. Actinomycetoma grains are smaller, softer and consist of filamentous bacteria. Cytologically, eumycetoma is identifiable by periodic acid–Schiff and Grocott-Gömöri methenamine silver stains, which highlight large fungal elements, and fine-needle aspiration cytology (FNAC) or imprint smears often show pigmented fungal colonies. Actinomycetoma grains stain homogenously eosinophilic on H&E and display a blue centre with a pink periphery on May–Grünwald–Giemsa staining; they are also Gram-positive. FNAC and imprint cytology are low-cost, reliable techniques for early differentiation, while special stains enhance fungal detection. Definitive diagnosis and species identification require culture.^4,5^

Actinomycetoma is caused by actinomycetes, primarily *Nocardia*, *Streptomyces* and *Actinomadura*. Their distribution varies by region: *Nocardia* is more common in Latin America, while *Streptomyces* predominates in Africa.^6^ Actinomycetomas generally progress more rapidly than eumycetomas and present with more inflammatory and destructive lesions. Vertebral body infections can lead to spinal cord compression, resulting in neurological complications, including paraplegia.^7^ While *N. brasiliensis* is the most common cause of actinomycetoma, accounting for 66–78% of cases, *N. vulneris* is an exceptionally rare pathogen.^8–10^ Only two cases of mycetoma caused by *N. vulneris* have been reported worldwide (Table 1).

**Table 1:** A literature review of mycetoma due to *Nocadia vulneris*

Reference	Patient	Clinical presentation	Biopsy findings	Cultures	PCR	Treatment
Sumioki^11^	Japanese man in his 30s	Painless nodules; progressed to ulcers, nodules, on right leg	First (ulcer): no malignancy Second (mass): *Nocardia*-like bacteria with sulfur granules, granulomatous response	No growth *Candida tropicalis*, *Candida parapsilosis*	*N. vulneris* not detected	MINO + TMP-SMX then MCFG + MINO/TMP-SMX; posaconazole
Eaton^1^	56-year-old Hispanic man	Painful, purulent drainage from posterior cervicothoracic lesions^a^	Tissue biopsies found neutrophilic clusters	Abscess fluid and tissue: *Nocardia* spp.	*N. vulneris*	TMP-SMX + meropenem, + amikacin; followed by moxifloxacin + TMP-SMX
Our case	32-year-old man from El Salvador	Painless nodules and purulent draining sinuses on back	Suppurative granuloma	*Nocardia* spp.	*N. vulneris*	TMP-SMX + dapsone

MCFG, micafungin sodium; MINO, minocycline; PCR, polymerase chain reaction; TMP-SMX, trimethoprim–sulfamethoxazole.

^a^Magnetic resonance imaging showned epidural abscesses, fistulous tracts, vertebral osteomyelitis and spinal cord compression; patient underwent laminectomy C7–T4, phlegmon removal, osseous biopsies.

Two cases of *N. vulneris* infection have been reported. In 2019, a 56-year-old man developed severe cervicothoracic disease with spinal involvement; diagnosis was confirmed via sequencing, and he was treated with antibiotics including lifelong TMP-SMX. In 2024, a healthy Japanese man developed leg nodules after a dog bite in Bali; molecular testing confirmed *N. vulneris*, and he recovered after a 9-month course of antifungals and antibiotics.^1,11^

Our case represents the first reported instance of actinomycetoma caused by *N. vulneris* on the back in Central America. *Nocardia* typically resides in soil, leading to primary cutaneous infections from penetrating injuries, most commonly affecting the foot. Studies show that the back is involved in approximately 8% of *Nocardia* mycetoma cases, compared with about 60% for the foot.^8,10^ Predisposing factors for actinomycetoma caused by *N. vulneris* remain unclear. However, *Nocardia* spp. infections are generally linked to direct inoculation with soil contaminated by the bacteria. Infection typically follows minor trauma from thorns, plants, or contaminated soil and tools, spreading locally or via lymphatics.^12^ Cutaneous infection may result from direct inoculation through trauma, surgery, insect bites or intralesional procedures, and can disseminate, particularly to the central nervous system.^13,14^ Even minor exposures, such as gardening injuries or contact with contaminated materials, can serve as entry points.^15,16^ In this case, the patient’s work in an egg factory may have led to back trauma from chicken scratches or contact with contaminated surfaces.

Mycetoma is a neglected tropical disease caused by over 70 microorganisms, but current diagnostic tools are limited, especially in affected regions. To improve care, the WHO developed two target product profiles: one to identify the causative organism and another to determine when treatment can safely stop. Ideally, tests should be quick, accurate (over 99% sensitivity) and identify specific organisms like *Nocardia* or *Madurella*; minimum standards accept lab-based tests that distinguish fungal from bacterial infections with at least 95% sensitivity and 75% specificity.^17^

For diagnosing actinomycetoma, the most common method is sequencing the 16S rDNA gene, which helps identify new species but struggles to differentiate closely related *Nocardia* strains. To overcome this, multilocus sequence analysis – which includes genes like *hsp65*, *gyrB*, *secA1* and *rpoB* – offers better accuracy. However, due to limited access to sequencing in endemic areas, PCR restriction fragment length polymorphism methods targeting genes like *16S rRNA*, *groEL* and *hsp65* have been developed using restriction enzymes (e.g. HaeIII, HinP1I, DpnII). These can distinguish most *Nocardia* species and even differentiate them from *Mycobacterium* species like *M. tuberculosis*. Faster identification is also possible using genus- or species-specific PCRs – such as a pan-*Nocardia* PCR which works directly on clinical samples.^6^

In our case, the diagnosis was confirmed through clinical presentation, direct examination of pus grains and 16S rDNA PCR sequencing, which identified *N. vulneris* from culture colonies. In comparison with two previously reported cases, the first identified *N. vulneris* via gene sequencing from culture colonies, while the second used 16S rDNA gene sequencing from tissue specimens. Definitive diagnosis should be confirmed by culturing grains or tissue on Sabouraud or blood agar, followed by PCR sequencing to identify the causative species.

TMP-SMX is the preferred first-line agent for localized *N. vulneris* infections, with reported success in combination with minocycline, imipenem, moxifloxacin or dapsone.^18,19^ For disseminated disease affecting the lungs, CNS or bone, combination therapy – typically with TMP-SMX, amikacin and meropenem – is recommended. A case of disseminated *N. vulneris* infection also responded well to a regimen of linezolid, amikacin and TMP-SMX, supporting its use in severe infections.^19,20^ In China, susceptibility rates for *Nocardia* spp. were highest for linezolid (99.5%), amikacin (96.0%) and TMP-SMX (92.9%), although variation exists across species. Linezolid and amikacin are consistently effective, particularly in resistant strains.^21^ A case of disseminated nocardiosis caused by *N. vulneris* was successfully treated with a combination of linezolid, amikacin and TMP-SMX. For disseminated nocardiosis, a minimum of 6–12 months of antimicrobial therapy is recommended.^20^

Advanced infection, more than dissemination alone, is linked to increased 1-year mortality in nocardiosis.^22^ Immunocompromised patients, particularly post–haematopoietic cell transplantation (HCT), are prone to severe, disseminated disease often involving the lungs, brain and bloodstream. Brain imaging is recommended in all HCT recipients with nocardiosis, regardless of neurological symptoms. Overall mortality remains high.^23^

Antimicrobial resistance in *Nocardia* species is species-specific and influenced by resistance genes or mutations. Linezolid is effective against all isolates, followed by TMP-SMX (93%) and amikacin (91%). Resistance mechanisms include aph(2″) in *N. farcinica* and *N. wallacei* (tobramycin resistance), blaAST-1 in *N. cyriacigeorgica* and *N. neocaledoniensis* (amoxicillin/clavulanate resistance), blaFAR-1 in *N. farcinica* (ceftriaxone resistance), Ser83Ala substitution in *gyrA* (ciprofloxacin resistance) and 16S rRNA m1A1408 methyltransferase in *N. wallacei* (amikacin resistance).^24^ In Spain, antimicrobial susceptibility analysis of the most prevalent *Nocardia* species showed that 82.9% of strains belonged to *N. cyriacigeorgica* (25.3%), *N. nova* (15.0%), *N. abscessus* (12.7%) and *N. farcinica* (11.4%), among others. Approximately 86% of isolates were obtained from the respiratory tract. Minimum inhibitory concentration results revealed notable variations between two time periods. Based on Clinical and Laboratory Standards Institute breakpoints, low resistance rates (≤15%) were observed in seven species for cefotaxime, imipenem and tobramycin, and in five species for TMP-SMX. Linezolid and amikacin remained the most consistently effective agents.^25^

In a retrospective study of 414 *Nocardia* isolates in Australia, molecular sequencing identified 27 species, predominantly from respiratory specimens (81.2%). *Nocardia nova* was most common (35.5%), followed by *N. ­cyriacigeorgica* (18.1%). *Nocardia farcinica* and *N. paucivorans* were more frequently isolated from sterile sites, while *N. brasiliensis* and *N. otitidiscaviarum* were associated with skin and soft tissue. Linezolid (100%) and amikacin (99%) showed the highest susceptibility rates, whereas ceftriaxone (59%) and imipenem (41%) were less effective.^26^

These findings underscore the importance of species identification and susceptibility testing for effective treatment. Cure rates were low: 12.7% for eumycetoma and 14.3% for actinomycetoma. Amputation was performed in 6.1% of cases of emycetoma, but in no cases of actinomycetoma. Predictors of cure included small lesion size [odds ratio (OR) 10.09, *P* < 0.001] and good follow-up (OR 6.81, *P* = 0.002). Risk factors for amputation included prior surgical recurrence (OR 3.67, *P* = 0.02) and presence of grains (OR 7.13, *P* = 0.01), while small lesions were protective (OR 0.06, *P* = 0.009).^27^ Overall, treatment outcomes were suboptimal, highlighting the need for earlier diagnosis and better management.^27,28^

In our case and two previous reports, combination therapy led to clinical improvement over a minimum 6-month course. One patient showed delayed response due to *Candida* co-­infection, highlighting the importance of addressing concurrent pathogens.

Another consideration is that if our patient shows signs of persistent infection, rebiopsy, PCR and culture are essential to assess for additional pathogen colonization or co-infection. For example, colonization by *Candida tropicalis* and *Candida parapsilosis* has been reported to delay clinical response. If no other pathogens are detected and *Nocardia* spp. remains the causative agent, alternative treatments may be considered, such as combining TMP-SMX with agents like imipenem, amikacin or meropenem. Surgical excision can also be an option for *Nocardia* mycetoma, particularly in patients with well-defined lesions or localized infections unresponsive to antibiotics alone. However, excision carries a potential risk of disseminated infection, as *Nocardia* spp. can spread haematogenously, especially in patients who are immunocompromised. Surgical manipulation could theoretically introduce organisms into the bloodstream, increasing the risk of dissemination.

Although *N. vulneris* actinomycetoma is rare, its occurrence highlights the importance of excluding disseminated infection. Molecular identification through 16S rDNA sequencing provides a rapid diagnostic tool and guides appropriate treatment. This case illustrates an uncommon presentation of *N. vulneris* mycetoma in an atypical location and demonstrates the efficacy of combined TMP-SMX and dapsone therapy without serious side effects.

## Data Availability

The data underlying this article will be shared on reasonable request to the corresponding author.
